# Precipitation Gradients Regulate Soil Carbon Stabilization Mediated by Microbes in an Arid Region of China

**DOI:** 10.3390/plants15203090

**Published:** 2026-10-09

**Authors:** Wen Li, Bing Liu, Arash Malekian, Changkun Yang, Weihao Sun, Bin Wang, Xiao Wang, Pouyan Dehghan Rahimabadi

**Affiliations:** 1 Linze Inland River Basin Research Station, State Key Laboratory of Ecological Safety and Sustainable Development in Arid Lands, China and Iran Joint Laboratory on Agriculture and Ecology in Arid Regions, Northwest Institute of Eco-Environment and Resources, Chinese Academy of Sciences, Lanzhou 730000, China; liwen24@mails.ucas.ac.cn (W.L.); yangchangkun@nieer.ac.cn (C.Y.); sunweihao22@nieer.ac.cn (W.S.); wangbin21@nieer.ac.cn (B.W.); wangxiao21@mails.ucas.ac.cn (X.W.); dehghan_r_p_12@ut.ac.ir (P.D.R.); 2 University of Chinese Academy of Sciences, Beijing 100049, China

**Keywords:** bacterial community, extracellular enzyme activity, mineral-associated organic matter, particulate organic matter

## Abstract

Desert ecosystems are important components of the terrestrial carbon cycle, yet the mechanisms by which altered precipitation regulates soil organic carbon (SOC) stabilization remain poorly understood. We evaluated long-term responses to precipitation manipulation in a desert shrub ecosystem in northwestern China. The experiment was established in 2011 with seven treatments ranging from −50% to +50% of ambient precipitation, and the comprehensive plant, soil, and microbial dataset analyzed in the present study was collected in 2025 after 15 consecutive growing seasons of manipulation. We examined plant growth traits, soil moisture, microbial biomass, extracellular enzyme activities, bacterial and fungal communities, and particulate and mineral-associated organic carbon fractions. Plant growth traits, microbial biomass, extracellular enzyme activities, and SOC fractions generally exhibited nonlinear responses across the precipitation gradient, whereas soil moisture increased with increasing precipitation. Moderate precipitation addition (+30%) enhanced microbial biomass and enzyme activity and significantly increased SOC, particulate organic carbon, and mineral-associated organic carbon by 92.5%, 90.5%, and 112.1%, respectively, relative to the control. However, these responses declined under the +50% treatment, indicating that further increases in water availability did not continuously promote carbon accumulation. Bacterial communities were more responsive to altered precipitation than fungal communities, while microbial α-diversity showed weaker relationships with carbon stability. Partial least squares structural equation modeling indicated that precipitation effects on carbon stability were associated primarily with changes in microbial biomass and extracellular enzyme activity; microbial biomass had the largest total effect, and both models explained 87% of the variation in carbon stability. These results suggest that SOC stabilization in this dryland shrub ecosystem responds nonlinearly to altered precipitation and is more closely associated with microbial functional processes than with microbial α-diversity.

## 1. Introduction

Desert ecosystems cover 13 to 33% of the terrestrial surface [1]. Despite their relatively low carbon density, their vast spatial extent and substantial soil carbon pools make them important components of the terrestrial carbon cycle. Understanding how desert soil carbon responds to climate change is therefore essential for predicting carbon–climate feedbacks [2,3,4]. Precipitation serves as the primary climatic factor that limits carbon cycling in desert ecosystems. Desert regions have extremely low precipitation and an uneven seasonal distribution. In conjunction with high potential evapotranspiration, these attributes synergistically constrain vegetation growth and reduce primary productivity, thereby substantially attenuating the carbon input capacity of ecosystems and collectively shaping the distinct carbon budget patterns of arid zones. In water-limited ecosystems, pulsed precipitation is a primary determinant of ecosystem structure and function because it regulates soil moisture and vegetation processes [5,6]. It also strongly affects microbial activity and organic matter turnover [7,8]. Long-term drought and aridification can also suppress microbial activity and decomposition processes, thereby slowing carbon turnover and further modulating the loss of soil carbon, with cascading impacts on biogeochemical cycling [9]. This precipitation-driven dynamic equilibrium of carbon budgets dominates the processes of carbon accumulation and turnover within desert ecosystems. Accordingly, the spatial heterogeneity and temporal fluctuations along precipitation gradients function as critical drivers that regulate carbon cycling in arid regions. Climate change projections suggest that global warming will significantly increase precipitation variability, thereby increasing the frequency and intensity of droughts and extreme precipitation events at interannual and longer timescales [10,11]. These shifts will restructure plant community composition and diversity, determine vegetation productivity and carbon input levels, and further alter soil carbon cycling through processes mediated by the structure and metabolic functions of soil microbial communities [12,13]. Thus, they will ultimately exert profound feedback effects on the regional and global carbon balance of desert ecosystems.

Recent studies have increasingly examined how precipitation variability affects microbial activity and vegetation-related carbon processes [14,15,16]. In parallel, research on soil organic carbon (SOC) has shifted from changes in total SOC stocks toward the regulation of carbon fractions and stabilization mechanisms [17,18,19]. Increasing evidence indicates that SOC persistence is determined not only by the quantity of carbon inputs but also, more importantly, by the allocation of carbon among functionally distinct pools [16,19]. Among these pools, particulate organic carbon (POC) and mineral-associated organic carbon (MAOC) represent two major fractions that govern SOC dynamics. POC is primarily derived from plant residues and partially decomposed organic matter and generally exhibits relatively rapid turnover, whereas MAOC represents a more persistent carbon pool formed mainly through microbial processing and subsequent association with mineral surfaces [20,21]. Therefore, the balance between POC and MAOC provides an important indicator of SOC lability and long-term stabilization.

Precipitation regulates transformation between SOC fractions by altering soil moisture conditions, substrate diffusion, and nutrient availability. Under increased precipitation, increased microbial activity can accelerate POC decomposition and promote the conversion of labile carbon into MAOC through microbial transformation pathways [22,23,24]. Conversely, reduced precipitation constrains microbial activity and substrate accessibility, leading to greater retention of POC in the SOC pool while limiting MAOC formation [18]. Thus, precipitation gradients influence SOC stability not only by regulating carbon decomposition rates but also by reshaping microbially mediated carbon transformation pathways. SOC fraction responses to precipitation changes are often nonlinear, and they often exhibit threshold effects, saturation responses, or asymmetric patterns [16,25,26]. These nonlinear responses reflect complex interactions among water availability, microbial metabolism, substrate supply, and mineral protection processes. Moderate precipitation increases may also increase MAOC formation by alleviating water limitations for microbes and stimulating microbial carbon processing; however, excessive precipitation may accelerate carbon mineralization and offset potential stabilization benefits [27]. In contrast, precipitation reduction can reduce microbially mediated carbon transformation and constrain the formation of stable carbon pools. However, it remains unclear how precipitation gradients regulate SOC stabilization in desert ecosystems, and the relative contributions of carbon decomposition and carbon-pool redistribution pathways across precipitation regimes remain poorly understood.

Microbial communities and extracellular enzymes serve as critical biological mediators that link precipitation variability to SOC stabilization. Key extracellular enzymes, including β-glucosidase (BG), *N*-acetyl-β-D-glucosaminidase (NAG), and phosphatase, regulate microbial carbon, nitrogen, and phosphorus acquisition processes [28,29]. These resource-acquisition processes can in turn influence microbial carbon–nitrogen dynamics and SOC stability [30,31]. However, microbial responses to altered precipitation vary among taxa because of differences in physiological traits and resource-acquisition strategies. Bacteria depend strongly on continuous water films for dispersal and substrate diffusion, whereas fungi can maintain resource acquisition under water stress through their extensive hyphal networks and dormant structures [32,33,34]. Consequently, bacterial communities often respond more strongly than fungal communities to changes in precipitation, with bacterial α-diversity declining under severe drought and recovering as water limitation is alleviated [35]. Precipitation effects on microbial communities are also mediated indirectly through environmental filtering associated with changes in soil water and nutrient availability [36,37]. Nevertheless, the long-term responses of bacterial and fungal communities across continuous precipitation gradients, and the extent to which microbial restructuring and enzyme activity regulate transformations between POC and MAOC, remain unclear.

To address these uncertainties, we conducted a long-term precipitation manipulation experiment in a desert shrub ecosystem with seven precipitation treatments: −50%, −30%, −10%, the control, +10%, +30%, and +50%. We quantified the responses of plant growth traits, soil water availability, microbial biomass, extracellular enzyme activities, bacterial and fungal communities, and SOC fractions across the precipitation gradient. Specifically, we aimed to: (1) characterize plant growth responses and determine how precipitation-induced variation in water availability affects microbial communities, enzyme activities, and SOC allocation between POC and MAOC; (2) assess whether SOC accumulation and stabilization exhibit nonlinear responses across the precipitation gradient; and (3) identify the principal microbial pathways associated with carbon transformation and stabilization.

Based on the strong water limitation characteristic of dryland ecosystems and the central role of microbial processing in SOC formation, we hypothesized that (1) alleviation of water limitation would initially enhance microbial biomass, extracellular enzyme activity, and the accumulation of both POC and MAOC, whereas excessive precipitation addition would weaken these responses, resulting in a nonlinear response across the precipitation gradient. We further hypothesized that (2) precipitation effects on SOC stabilization would be associated more strongly with microbial biomass and extracellular enzyme activity than with microbial α-diversity, and that bacterial communities would exhibit greater sensitivity to altered precipitation than fungal communities. By integrating long-term precipitation manipulation with microbial and SOC-fraction analyses, our study provides a mechanistic framework for understanding dryland soil carbon responses to future precipitation change.

## 2. Results

### 2.1. Responses of Plant Growth Traits to Altered Precipitation

**Figure 1:**
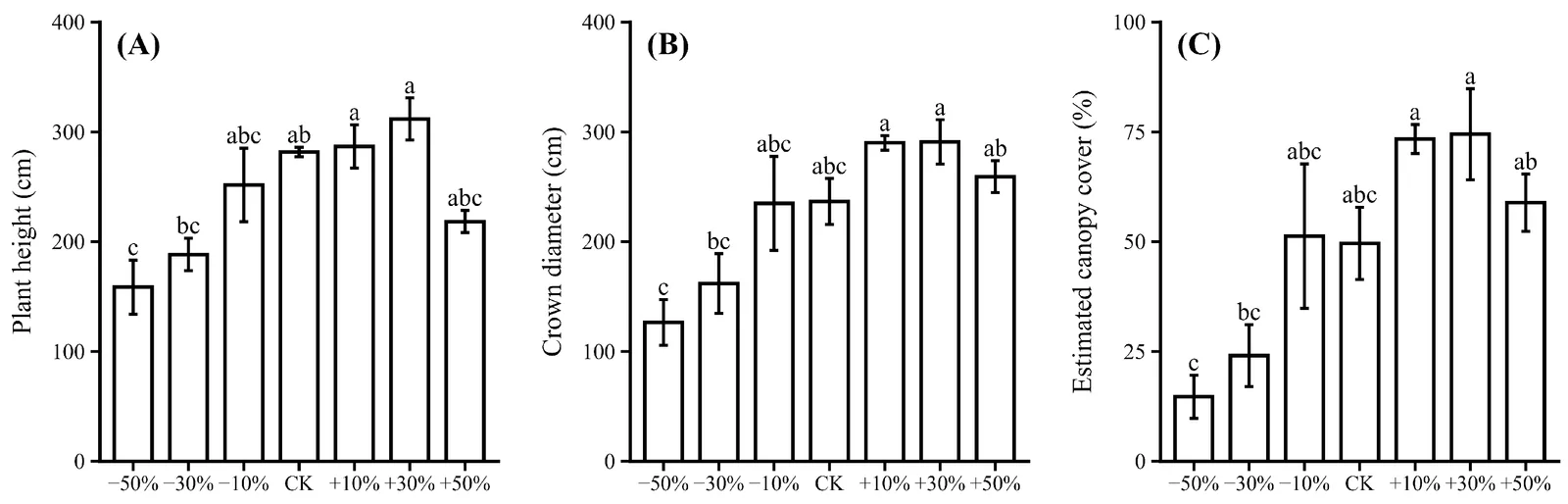
Responses of *Caragana korshinskii* growth traits to altered precipitation. (**A**) Plant height, (**B**) crown diameter, and (**C**) estimated canopy cover across the seven precipitation treatments. Crown diameter was calculated as the mean of two perpendicular crown diameters, and canopy cover was estimated from the projected elliptical crown area relative to the 9-m^2^ plot area. Values are means ± SE (*n* = 3). Different lowercase letters above the bars indicate significant differences among treatments based on one-way ANOVA followed by Tukey’s HSD test (*p* < 0.05); bars sharing the same letter are not significantly different.

Plant growth traits of *Caragana korshinskii* varied significantly across the precipitation treatments (Figure 1). Plant height increased from the precipitation-reduction treatments to a maximum under +30% precipitation and subsequently declined under +50% precipitation (Figure 1A). Plant height under +30% precipitation was significantly greater than that under the −50% and −30% treatments. Crown diameter showed a similar response pattern (Figure 1B), increasing from 126.5 cm under −50% precipitation to 290.8 cm under +30% precipitation, with a similarly high value under +10% precipitation (290.0 cm). Crown diameter under the +10% and +30% treatments was significantly greater than that under the −50% and −30% treatments, whereas the mean value declined under +50% precipitation. Estimated canopy cover also differed significantly among treatments (Figure 1C), increasing from 14.7% under −50% precipitation to 73.4% and 74.5% under the +10% and +30% treatments, respectively. Canopy cover under +10% and +30% precipitation was significantly greater than that under the −50% and −30% treatments, but declined to 58.9% under +50% precipitation. Overall, severe precipitation reduction constrained shrub growth, whereas moderate precipitation addition promoted plant height, crown expansion, and canopy development; these responses did not continue to increase under the highest precipitation-addition treatment.

### 2.2. Responses of SMC, Microbial Biomass, and Extracellular Enzyme Activities to Altered Precipitation

**Figure 2:**
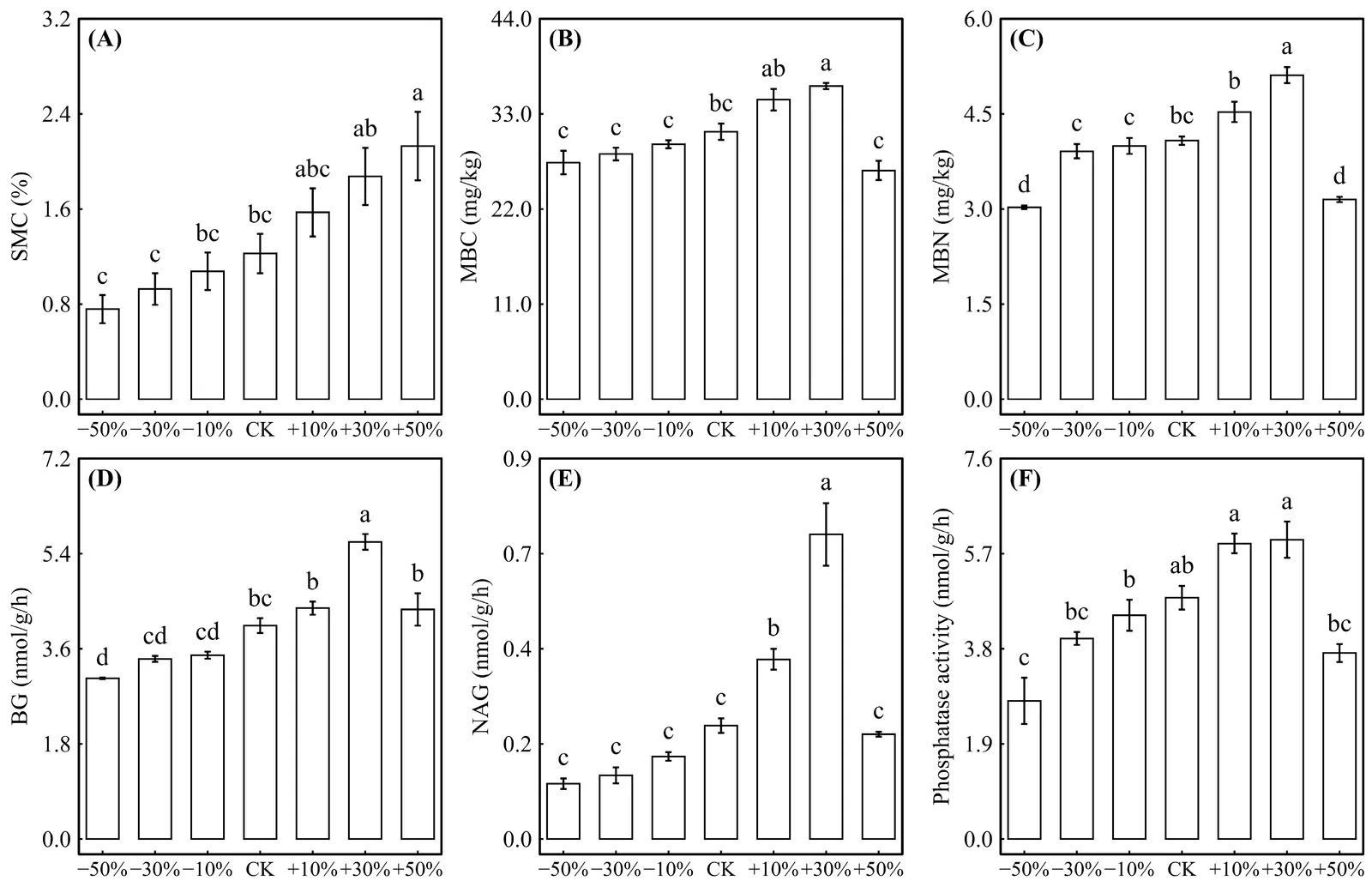
Soil moisture content (SMC), microbial biomass (MBC and MBN), and extracellular enzyme activities under the different precipitation treatments. (**A**) Soil moisture content (SMC; %); (**B**) microbial biomass carbon (MBC; mg kg^−1^); (**C**) microbial biomass nitrogen (MBN; mg kg^−1^); (**D**) β-glucosidase activity (BG; nmol g^−1^ h^−1^); (**E**) *N*-acetyl-β-D-glucosaminidase activity (NAG; nmol g^−1^ h^−1^); and (**F**) phosphatase activity (nmol g^−1^ h^−1^) across seven precipitation treatments. Values are means ± SE (*n* = 3). Different lowercase letters above the bars indicate significant differences among treatments based on one-way ANOVA followed by Tukey’s HSD test (*p* < 0.05); bars sharing the same letter are not significantly different.

Soil moisture content increased with increasing precipitation, although only the +50% treatment was significantly higher than the control (Figure 2A). In contrast, microbial biomass and extracellular enzyme activities showed nonlinear responses. Microbial biomass carbon and nitrogen peaked under +30% precipitation, increasing by 17.1% and 25.3%, respectively, relative to the control, but declined under +50% precipitation (Figure 2B,C). Similarly, β-glucosidase, *N*-acetyl-β-D-glucosaminidase, and phosphatase activities generally increased toward +30% precipitation and subsequently decreased under +50% precipitation (Figure 2D–F). Severe precipitation reduction also suppressed microbial biomass and enzyme activities, with the strongest effects generally occurring under −50% precipitation. Overall, microbial biomass and enzyme activity exhibited unimodal responses despite the continued increase in soil moisture.

### 2.3. Responses of SOC Fractions to Altered Precipitation

**Figure 3:**
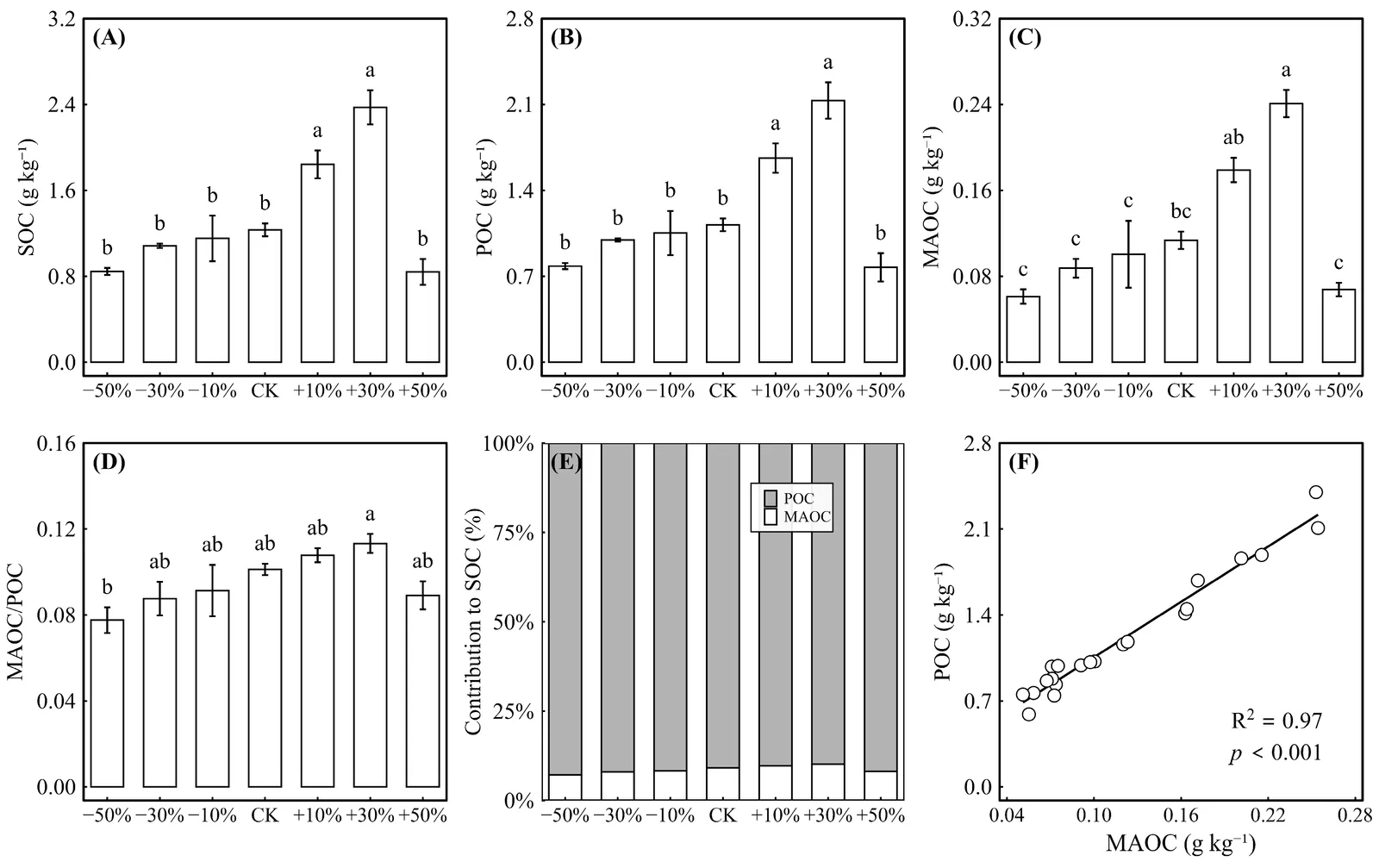
Soil organic carbon fractions and their relative allocation under different precipitation treatments. (**A**) Soil organic carbon (SOC; g kg^−1^); (**B**) particulate organic carbon (POC; g kg^−1^); (**C**) mineral-associated organic carbon (MAOC; g kg^−1^); (**D**) the MAOC/POC ratio; (**E**) the relative contributions of POC and MAOC to SOC (%); and (**F**) the relationship between MAOC and POC across the individual samples. Values are means ± SE (*n* = 3). Different lowercase letters indicate significant differences among precipitation treatments based on one-way ANOVA followed by Tukey’s HSD test (*p* < 0.05). The solid line in panel F represents the fitted linear regression (R^2^ = 0.97, *p* < 0.001).

Soil organic carbon fractions responded nonlinearly to altered precipitation (Figure 3). Relative to the control, +30% precipitation significantly increased SOC, POC, and MAOC by 92.5%, 90.5%, and 112.1%, respectively (Figure 3A–C). SOC and POC under +10% and +30% precipitation were significantly higher than under +50%, whereas the precipitation-reduction treatments and +50% did not differ significantly from the control. The MAOC/POC ratio varied less strongly across treatments, with its highest value under +30% precipitation (Figure 3D). POC accounted for 89.85–92.77% of the measured SOC fractions, whereas MAOC contributed 7.23–10.15% (Figure 3E). POC and MAOC were strongly positively related across individual samples (R^2^ = 0.97, *p* < 0.001; Figure 3F). Overall, moderate precipitation addition enhanced both carbon fractions, whereas further addition to +50% reversed these increases.

### 2.4. Responses of Bacterial and Fungal Communities to Altered Precipitation

#### 2.4.1. Responses of Bacterial and Fungal α-Diversity to Altered Precipitation

**Figure 4:**
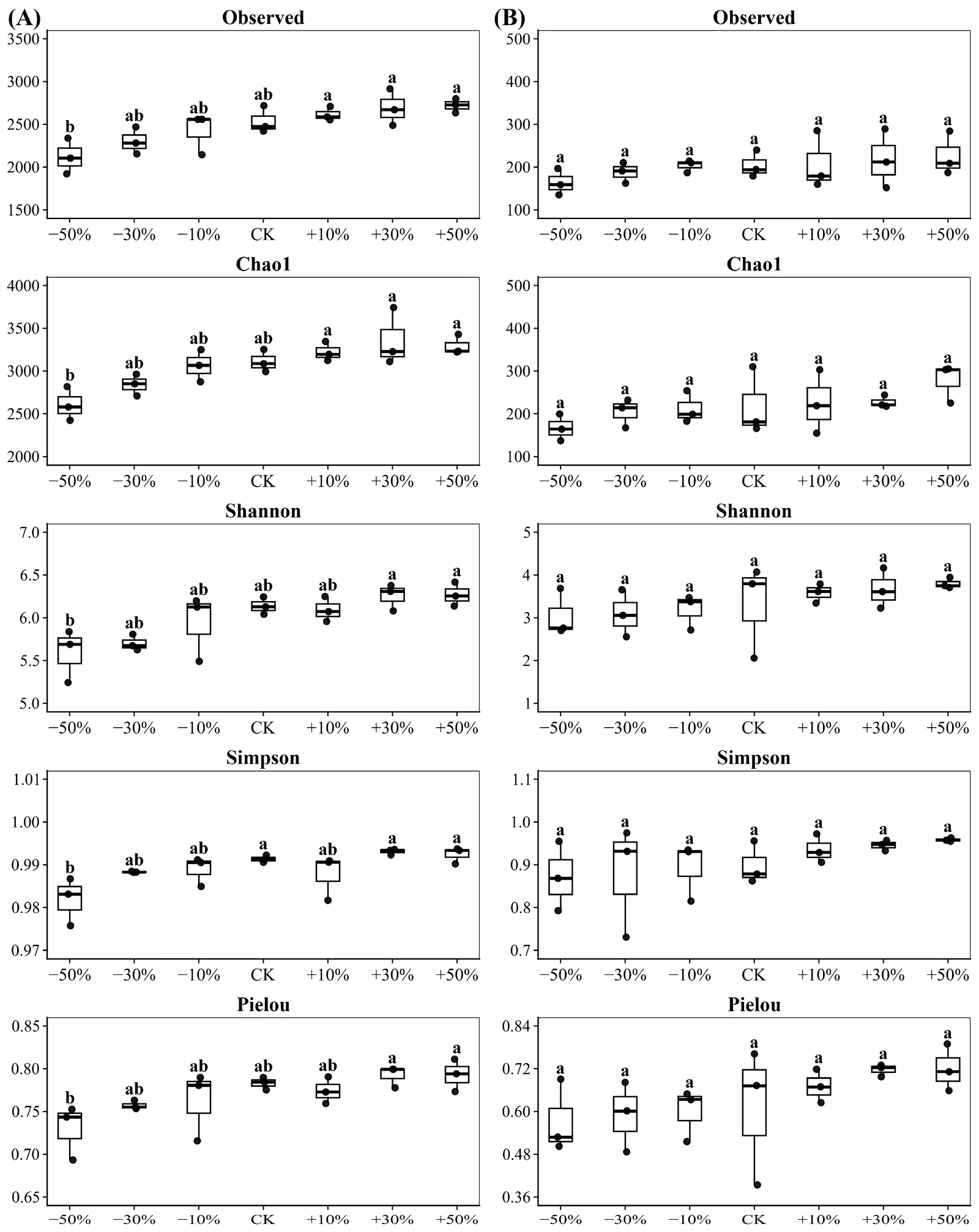
Responses of bacterial and fungal α-diversity to altered precipitation. Boxplots show Observed richness, Chao1 richness, Shannon diversity, Simpson diversity, and Pielou evenness for (**A**) bacterial and (**B**) fungal communities across the seven precipitation treatments. The horizontal black line within each box indicates the median, boxes represent the interquartile range, whiskers indicate the spread of the data, and black points represent individual replicates (*n* = 3). Different lowercase letters indicate significant differences among treatments based on one-way ANOVA followed by Tukey’s HSD test (*p* < 0.05).

Bacterial α-diversity was more responsive to altered precipitation than fungal α-diversity (Figure 4). Relative to the control, −50% precipitation reduced bacterial Observed richness, Chao1 richness, Shannon diversity, and Pielou evenness by 16.39%, 16.20%, 8.93%, and 6.81%, respectively. Observed and Chao1 richness under −50% precipitation were significantly lower than those under +10%, +30%, and +50%, whereas Shannon diversity and Pielou evenness were significantly lower than those under +30% and +50%. In contrast, Observed richness, Shannon diversity, and Pielou evenness reached their highest values under +50% precipitation, increasing by 7.19%, 2.15%, and 1.25% relative to the control, respectively, whereas Chao1 richness peaked under +30% precipitation, increasing by 7.97%.

Fungal α-diversity showed no significant differences among precipitation treatments (Figure 4B), despite numerical variation across the precipitation gradient. Relative to the control, −50% precipitation numerically reduced fungal Observed richness, Chao1 richness, and Shannon diversity by 19.90%, 23.78%, and 7.85%, respectively, whereas +50% precipitation increased these indices numerically by 10.93%, 26.79%, and 14.86%, respectively. However, none of the fungal α-diversity indices differed significantly among treatments.

#### 2.4.2. Taxonomic and Phylogenetic Responses of Bacterial and Fungal Communities to Altered Precipitation

**Figure 5:**
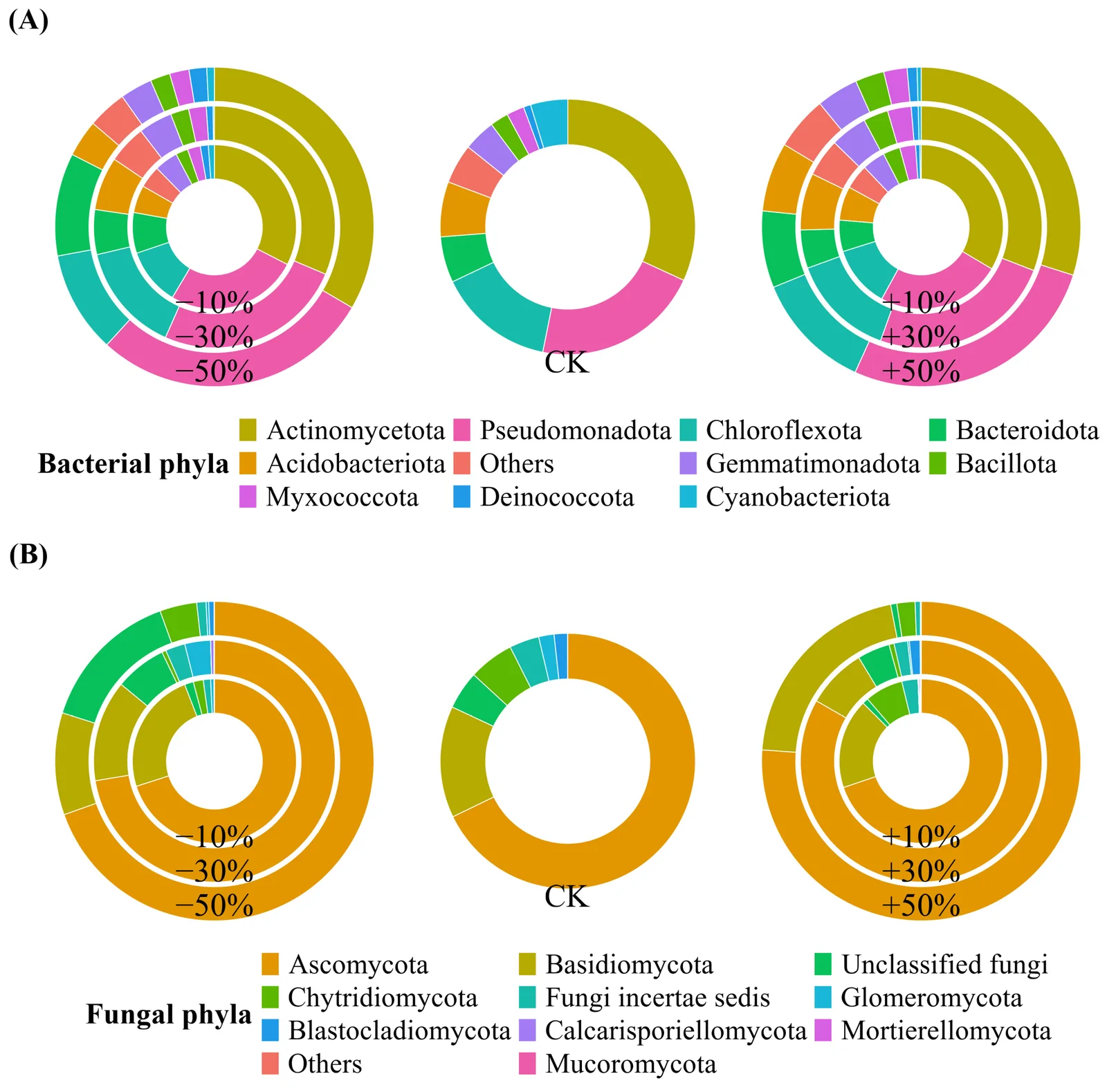
Phylum-level composition of bacterial and fungal communities under altered precipitation. Relative abundance of the dominant phyla in (**A**) bacterial and (**B**) fungal communities across the seven precipitation treatments (−50%, −30%, −10%, control, +10%, +30%, and +50%). Relative abundances are expressed as percentages of the total bacterial or fungal sequences within each treatment.

Bacterial and fungal communities showed contrasting phylum-level responses to altered precipitation (Figure 5). Bacterial communities were dominated by Actinomycetota, Pseudomonadota, Chloroflexota, Bacteroidota, and Acidobacteriota, which together accounted for approximately 83% of the community. Pseudomonadota generally increased under precipitation manipulation, whereas Chloroflexota decreased, with the largest compositional shifts occurring under severe precipitation reduction (Figure 5A). Fungal communities were dominated by Ascomycota and Basidiomycota, which together represented the majority of fungal sequences (Figure 5B). Although their relative abundances varied among treatments, Ascomycota remained dominant across the precipitation gradient. Overall, bacterial taxonomic composition showed greater treatment-associated variation than fungal composition.

Phylogenetic distributions of the dominant bacterial and fungal OTUs are provided in Figures S1 and S2.

### 2.5. Microbial–Environment Relationships and Pathways to SOC Stability

#### 2.5.1. Environmental Correlates of Bacterial and Fungal Community Composition

**Figure 6:**
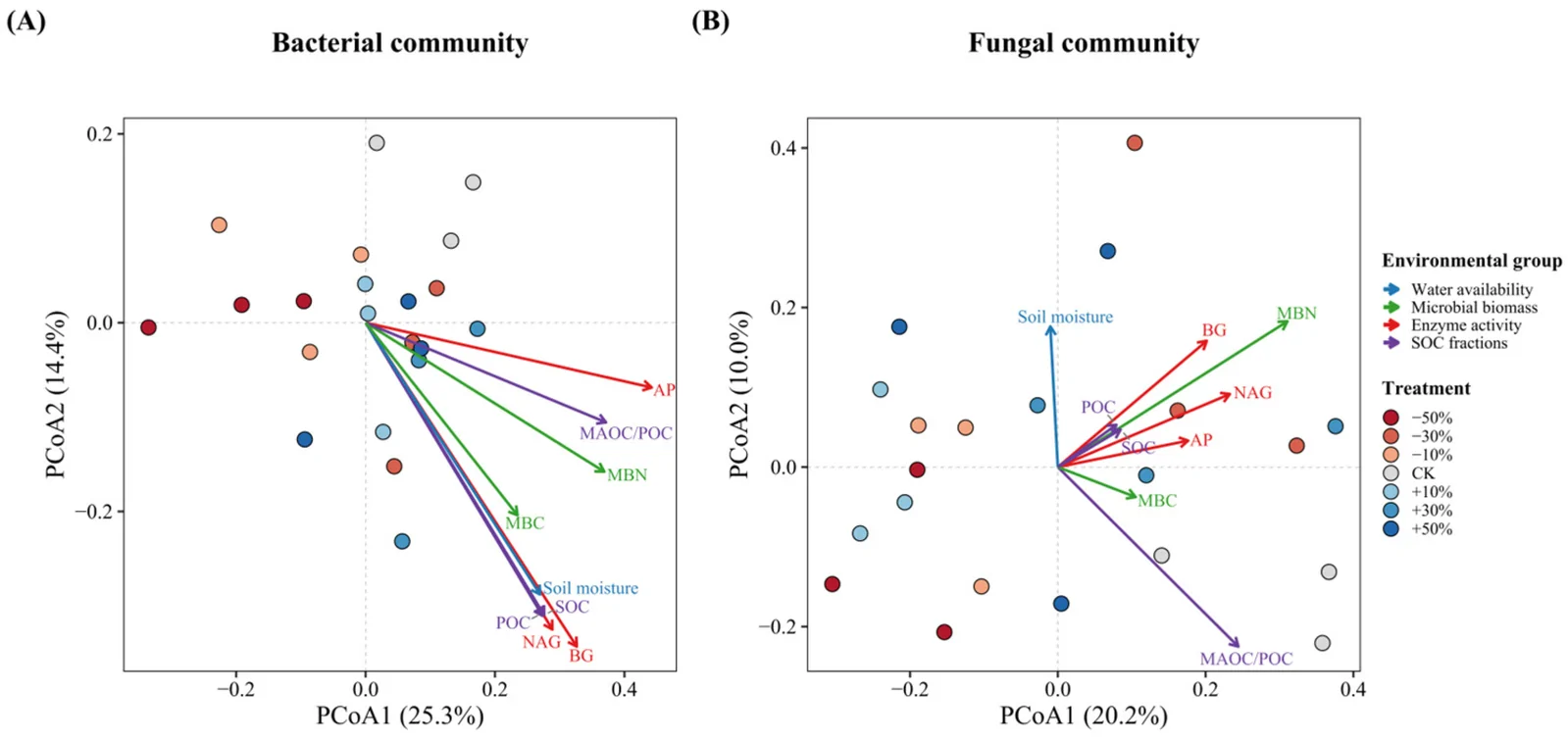
Relationships between the microbial community composition and environmental variables under altered precipitation. Principal coordinates analysis (PCoA) based on Bray–Curtis dissimilarities shows the composition of (**A**) bacterial and (**B**) fungal communities across seven precipitation treatments (−50%, −30%, −10%, control, +10%, +30%, and +50%). Points represent individual soil samples, and colors indicate the precipitation treatments. Environmental variables were fitted to each ordination using the envfit function with 999 permutations. Arrow direction indicates the direction of the association, whereas arrow length represents its relative strength. Arrow colors distinguish the soil moisture content (SMC), microbial biomass (MBC, microbial biomass carbon; MBN, microbial biomass nitrogen), enzyme activities (BG, β-glucosidase; NAG, *N*-acetyl-β-D-glucosaminidase; AP, phosphatase activity), and SOC fractions (MAOC, mineral-associated organic carbon; POC, particulate organic carbon; MAOC/POC). Axis labels indicate the percentage of variation explained by each PCoA axis. The dashed gray horizontal and vertical lines indicate zero on the PCoA2 and PCoA1 axes, respectively, and are included as visual reference guides.

PCoA revealed contrasting community–environment relationships between bacteria and fungi under altered precipitation (Figure 6). The first two axes explained 39.7% and 30.2% of the variation in bacterial and fungal community composition, respectively. Envfit analysis identified significant but moderate associations between bacterial community composition and β-glucosidase (BG; R^2^ = 0.36, *p* = 0.02), phosphatase activity (AP; R^2^ = 0.32, *p* = 0.03), *N*-acetyl-β-D-glucosaminidase (NAG; R^2^ = 0.30, *p* = 0.04), SOC (R^2^ = 0.28, *p* = 0.04), and POC (R^2^ = 0.27, *p* = 0.04) (Table 1). In contrast, none of the measured environmental variables was significantly associated with fungal community composition (all *p* > 0.05). These results indicate that bacterial community variation was more closely associated with enzyme activities and soil carbon pools than fungal community variation.

#### 2.5.2. PLS-SEM Analysis of Direct and Indirect Pathways Linking Precipitation to Carbon Stability

**Figure 7:**
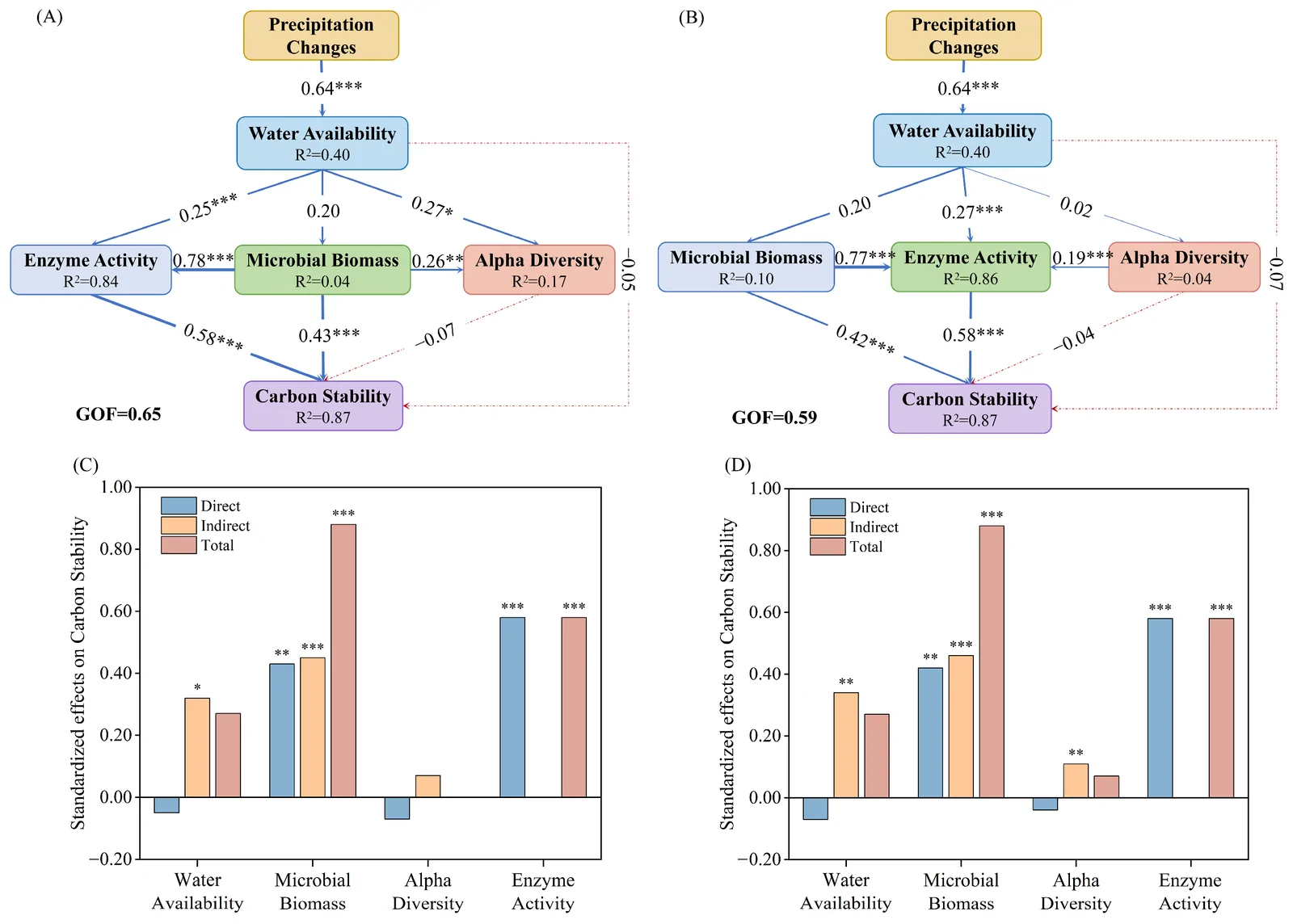
Partial least squares structural equation modeling of the pathways that link altered precipitation to carbon stability. The models show the (**A**) bacterial and (**B**) fungal pathways linking altered precipitation, soil water availability, microbial biomass, α-diversity, enzyme activity, and carbon stability. Numbers beside the arrows represent standardized path coefficients (β). Solid arrows indicate significant paths, whereas dashed arrows indicate nonsignificant paths; blue and orange arrows denote positive and negative effects, respectively. R^2^ values indicate the proportion of variance explained in the endogenous latent variables. Panels (**C**,**D**) show the direct, indirect, and total effects of each predictor on carbon stability in the bacterial and fungal models, respectively. GOF values indicate the overall goodness of fit of the models. Significance levels are ***, *p* < 0.001; **, *p* < 0.01; and *, *p* < 0.05.

PLS-SEM revealed similar pathways linking altered precipitation to carbon stability in the bacterial and fungal models (Figure 7). Precipitation positively affected water availability (β = 0.64), which subsequently influenced microbial functional processes. Microbial biomass strongly promoted enzyme activity in both models (β = 0.78 and 0.77 for bacteria and fungi, respectively), and enzyme activity had the strongest direct positive effect on carbon stability (β = 0.58, *p* < 0.001). Microbial biomass exerted the largest total effect on carbon stability (0.88), whereas α-diversity had no significant direct effect in either model. Both models explained 87% of the variation in carbon stability. Overall, precipitation effects on carbon stability were transmitted primarily through changes in microbial biomass and enzyme activity rather than through microbial α-diversity.

## 3. Discussion

### 3.1. Precipitation-Driven Changes in Plant Growth, Soil Microbial Activity and Carbon Fractions

Altered precipitation affected both plant growth and belowground microbial and carbon processes in this dryland shrub ecosystem. Plant height, crown diameter, and estimated canopy cover generally increased as the severe water limitation was alleviated, reaching their highest or near-highest values under moderate precipitation addition before declining under the +50% treatment (Figure 1). This pattern indicates that long-term precipitation alteration affected not only belowground processes but also the growth and canopy development of *C. korshinskii*. The broadly similar nonlinear responses of plant growth traits, microbial biomass, extracellular enzyme activities, and SOC fractions suggest coordinated above- and belowground sensitivity to altered water availability. Moderate precipitation addition increased microbial biomass and extracellular enzyme activities, whereas these responses declined under the highest precipitation-addition treatment (Figure 2B–F). These coordinated responses indicate that alleviating water limitation increased microbial growth and resource acquisition. In dryland soils, greater water availability can improve substrate diffusion and microbial physiological activity [38,39]. Improved soil moisture conditions can also increase enzyme–substrate contact, thereby stimulating microbial metabolism and organic matter processing [40]. SOC fractions exhibited similarly nonlinear responses, with SOC, POC, and MAOC increasing under moderate precipitation addition and declining under the highest addition treatment (Figure 3A–C). The comparatively strong response of MAOC suggests that moderate precipitation not only increased carbon accumulation but also favored MAOC accumulation [41]. Such differences are consistent with the distinct formation, turnover, and persistence mechanisms of POC and MAOC [16,19]. MAOC formation is particularly dependent on microbial processing and subsequent association of microbial-derived carbon with mineral surfaces [42]. However, these positive responses were reversed under the highest precipitation addition treatment, accompanied by marked decreases in microbial biomass and enzyme activities (Figure 2B–F and Figure 3A–D). This coordinated reversal indicates a nonlinear response with an apparent optimum near +30% rather than a monotonic response to increasing precipitation, which is consistent with the nonlinear and asymmetric responses reported in other water-limited ecosystems [43,44,45]. Overall, these nonlinear responses of microbial biomass, extracellular enzyme activity, POC, and MAOC are consistent with the first hypothesis. Moderate wetting likely alleviated a biological water limitation, whereas further water addition may have altered the balance among carbon inputs [45], microbial processing, and carbon retention. Nevertheless, the measured plant traits characterize shrub growth and canopy development but do not directly quantify plant-derived carbon inputs. Because above- and belowground biomass production, root turnover, litter inputs, soil respiration, and plant physiological responses were not directly measured, the contribution of vegetation-derived carbon to the observed changes in SOC fractions remains uncertain.

The differential responses of POC and MAOC further emphasize the importance of distinguishing functionally different SOC pools rather than considering bulk SOC alone [18,46]. POC is generally associated with partially decomposed plant-derived material, whereas MAOC largely forms through microbial processing of organic matter and subsequent association with mineral surfaces [19]. In our study, POC and MAOC showed a strong positive relationship across the precipitation gradient (Figure 3F), indicating coordinated variation in these two carbon pools. Moderate precipitation may have increased the supply of relatively labile substrates for microbial processing and subsequent MAOC accumulation, whereas further precipitation addition appears to have reduced the net retention of both pools. Similar differences in the sensitivity of SOC fractions to altered water availability have been reported in other water-limited ecosystems [47,48]. Collectively, these results indicate that precipitation regulates SOC stabilization through a nonlinear balance among microbial activity, carbon processing, and the accumulation and retention of distinct SOC fractions.

### 3.2. Asymmetric Responses of Bacterial and Fungal Communities to Altered Precipitation

Bacterial and fungal communities exhibited markedly asymmetric responses to long-term precipitation alteration. Severe precipitation reduction constrained bacterial α-diversity relative to precipitation-addition treatments, whereas alleviating water limitation promoted its recovery (Figure 4A). Bacterial communities in semiarid ecosystems are known to respond sensitively to precipitation variation [49]. This sensitivity is partly related to their dependence on continuous water films for dispersal, nutrient diffusion, and substrate acquisition [50]. Consequently, reductions in soil moisture can restrict bacterial activity and resource acquisition under strongly water-limited conditions [51,52]. This interpretation is also consistent with evidence that bacterial communities are generally less resistant to drought than fungal communities [53]. Notably, bacterial α-diversity did not differ significantly among the control and precipitation-addition treatments, despite pronounced decreases in microbial biomass and enzyme activities from +30% to +50%. Thus, bacterial diversity remained relatively high even when microbial functioning declined, indicating a partial decoupling between taxonomic diversity and functional activity [54,55].

In contrast, fungal α-diversity showed no significant response across the precipitation gradient (Figure 4B). The lack of a significant fungal α-diversity response may partly reflect the ability of fungal hyphae to bridge spatially isolated resource patches and sustain resource acquisition under dry conditions [56]. Fungi also generally exhibit greater resistance to severe water stress than bacteria [53,57]. Taxonomic patterns were consistent with this contrast. Bacterial communities were distributed among several dominant phyla and exhibited greater treatment-dependent variation, whereas fungal communities remained strongly dominated by Ascomycota and retained a comparatively conserved structure (Figure 5A,B). The phylogenetic distributions of the dominant bacterial and fungal OTUs showed similar contrasting patterns (Figures S1 and S2). These findings indicate that bacteria were more responsive to precipitation-induced environmental change, whereas fungi maintained greater compositional stability under fluctuating moisture conditions [34,49].

### 3.3. Linkages Between Microbial Community Composition, Enzyme Activity, and Carbon Dynamics

The contrasting bacterial and fungal responses were further reflected in their associations with soil biochemical processes. Among the variables fitted to bacterial community composition, extracellular enzyme activities and soil carbon pools showed significant but moderate associations (Figure 6A; Table 1). These associations were stronger than the association with soil moisture content itself, suggesting that bacterial community reorganization was more closely linked to microbial resource-acquisition processes and associated biochemical changes than to water availability alone. The associations of β-glucosidase, *N*-acetyl-β-D-glucosaminidase, and phosphatase activity with bacterial community composition indicate coupling between community restructuring and microbial C-, N-, and P-acquisition processes [28,58]. The association with β-glucosidase further emphasizes the potential importance of C-acquiring processes in linking bacterial community shifts to SOC turnover [28,59]. SOC and POC were also significantly associated with bacterial community composition, whereas the MAOC/POC ratio showed a weaker relationship (Figure 6A; Table 1). This pattern suggests a more immediate linkage between bacterial communities and bulk or relatively labile carbon pools, whereas changes in carbon stabilization may emerge through cumulative microbial processing and mineral association. However, the moderate magnitude of these associations indicates that the measured variables explained only part of the variation in bacterial community composition. Therefore, additional environmental and biological factors not measured in the present study may also contribute to bacterial community variation.

By contrast, none of the measured variables was significantly associated with fungal community composition (Figure 6B; Table 1). This weaker environmental coupling is consistent with the limited fungal α-diversity response and comparatively stable taxonomic structure. Fungal responses may also depend on factors not fully captured in the present study, including plant-derived carbon inputs, plant–fungal associations, litter quality, and microsite heterogeneity [36,49]. Overall, bacterial community reorganization was more closely associated than fungal restructuring with enzyme-mediated nutrient acquisition and SOC dynamics, further supporting a stronger functional coupling between bacteria and precipitation-induced changes in the soil biochemical environment.

### 3.4. Microbial Biomass and Enzyme Activity Link Precipitation to Carbon Stability

The PLS-SEM analysis integrated the abovementioned responses into a hierarchical pathway that linked altered precipitation to SOC stability (Figure 7A,B). Precipitation increased water availability, which subsequently influenced downstream microbial processes. In both the bacterial and fungal models, microbial biomass was strongly associated with enzyme activity, indicating that precipitation effects were transmitted from changes in the soil water environment to microbial functional processes rather than through a simple direct moisture effect. Extracellular enzyme activity exerted the strongest direct positive effect on carbon stability, whereas microbial biomass showed the largest total effect; both models explained a high proportion of the variation in carbon stability (Figure 7A–D). Microbial biomass may contribute to SOC stabilization through biomass turnover and subsequent microbial-derived carbon inputs [60]. Extracellular enzymes regulate substrate decomposition and microbial resource acquisition, thereby providing an important functional link between microbial activity and SOC transformation [28,59]. These mechanisms provide a plausible interpretation of the strong biomass–enzyme–carbon stability pathway identified by the PLS-SEM, although microbial necromass was not directly measured in the present study. In contrast, α-diversity had no significant direct effect on carbon stability in either the bacterial or fungal model (Figure 7A,B). This result is consistent with the divergence between microbial diversity and functioning observed across the precipitation gradient. Although bacterial α-diversity remained relatively high once severe water limitation was alleviated, microbial biomass and enzyme activity declined under the highest precipitation-addition treatment. Thus, greater taxonomic diversity did not necessarily correspond to stronger microbial functioning or greater SOC stabilization, indicating a partial decoupling between microbial taxonomy and function [54].

Overall, the PLS-SEM supports a conceptual pathway in which altered precipitation modifies soil water availability, which is subsequently associated with microbial biomass and extracellular enzyme activity and ultimately with carbon stability (Figure 7). These relationships should be interpreted as integrated statistical pathways rather than definitive evidence of causality. Together, the stronger associations of carbon stability with microbial biomass and extracellular enzyme activity than with microbial α-diversity, combined with the greater precipitation sensitivity of bacterial than fungal communities, are consistent with the second hypothesis.

## 4. Materials and Methods

### 4.1. Study Area and Climatic Background

**Figure 8:**
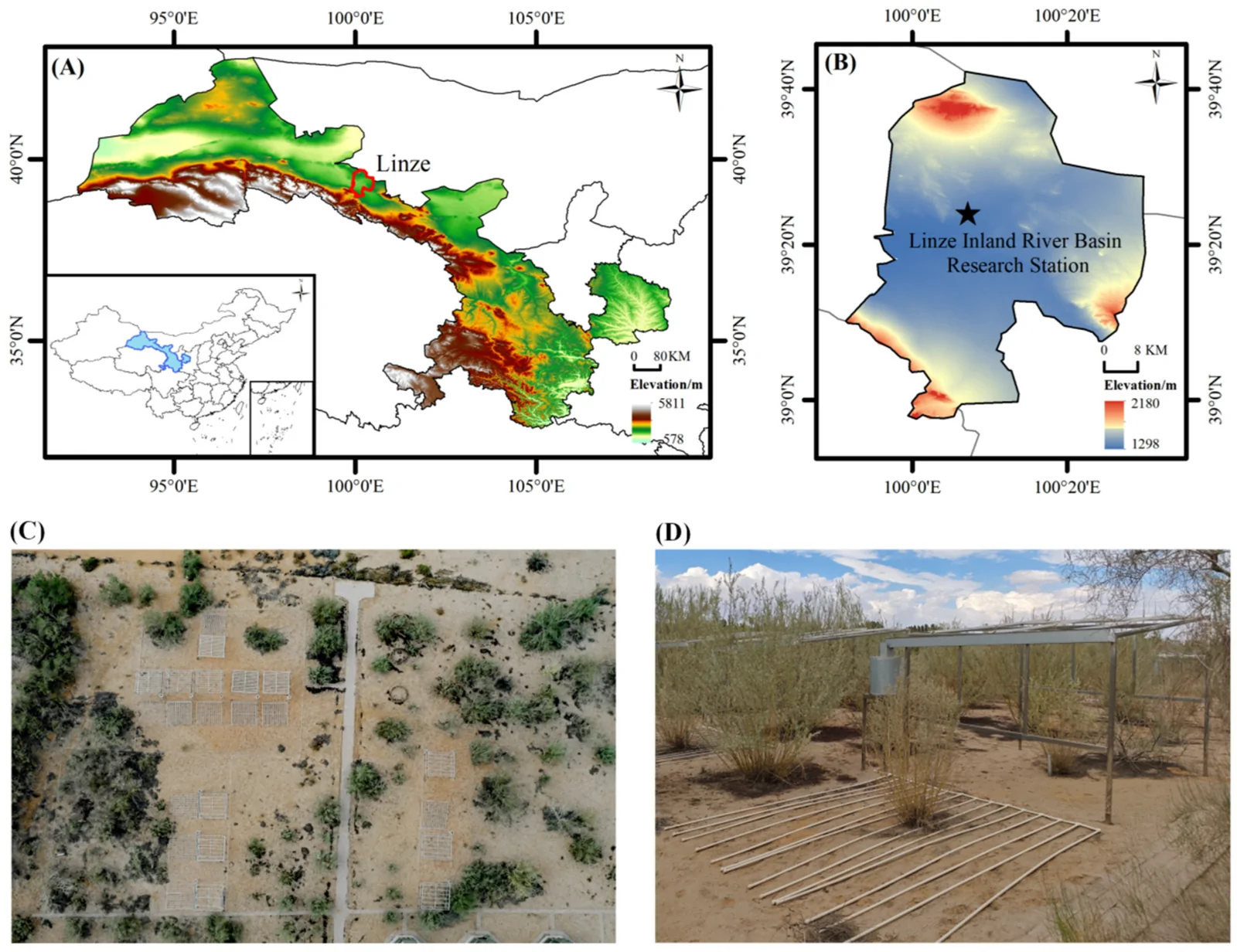
Geographic location and experimental layout. (**A**) Location of the study area in Gansu Province, northwestern China. (**B**) Elevation map showing the location of the Linze Inland River Basin Research Station. The black star indicates the location of the Linze Inland River Basin Research Station. (**C**) Aerial photograph of the experimental plots. (**D**) Field photograph showing the precipitation manipulation shelters in the dryland shrub ecosystem.

Our study was conducted at the Linze Inland River Basin Research Station, located in the arid region of northwestern China (39°21′ N, 100°02′ E; altitude: 1400 m; Figure 8A,B). The site is characterized by a typical temperate continental climate, with hot, dry summers and cold winters. The mean annual temperature is approximately 8.5 °C, with monthly mean temperatures ranging from −12.3 °C in January to 28.7 °C in July. The frost-free period lasts approximately 150 days per year. Mean annual precipitation is 117 mm and is primarily concentrated during the summer months (June–August), whereas winter precipitation accounts for only a small fraction of the annual total. Potential evapotranspiration is 2390 mm, reflecting the arid climatic conditions of the region. The vegetation is dominated by shrub species, including *Haloxylon ammodendron*, *Nitraria sphaerocarpa*, *Tamarix chinensis*, and *Caragana korshinskii*. The soils are predominantly sandy and are classified as Aridisols, consistent with the arid environmental conditions of the region.

**Figure 9:**
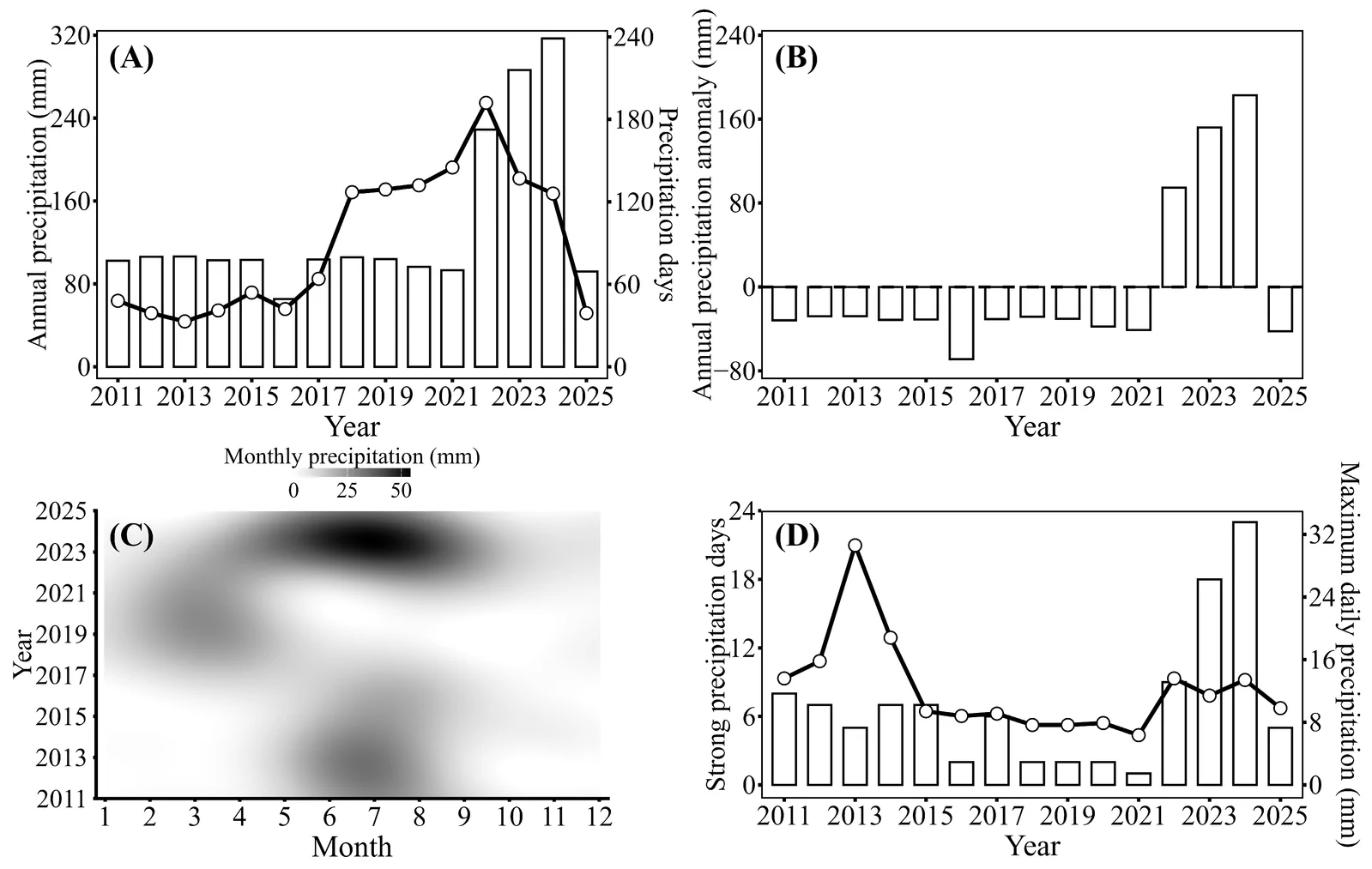
Interannual variation in precipitation amount and precipitation-event characteristics from 2011 to 2025. (**A**) Annual precipitation and number of precipitation days. Bars represent annual precipitation, and the line represents the annual number of precipitation days. (**B**) Annual precipitation anomalies relative to the mean from 2011 to 2025. (**C**) Generalized additive model (GAM)-smoothed distribution of monthly precipitation across years. (**D**) Annual number of strong precipitation days and maximum daily precipitation. Bars represent the number of strong precipitation days, and the line represents maximum daily precipitation. A precipitation day was defined as a day with precipitation > 0 mm, whereas a strong precipitation day was defined as a day with precipitation ≥ 5 mm.

During the experimental period (2011–2025), annual precipitation exhibited substantial interannual variability (Figure 9). Precipitation was relatively low during 2011–2021, followed by a pronounced wet period during 2022–2024, when annual precipitation ranged from 228.9 to 316.9 mm, before declining again in 2025. Precipitation was strongly seasonal, with 61.2% of the annual total occurring from May to August. The wet period during 2022–2024 was mainly characterized by a greater frequency of strong precipitation events rather than exceptionally high individual rainfall events.

### 4.2. Experimental Design

In 2011, the experimental site was an unvegetated natural desert. At the beginning of the experiment, one-year-old seedlings of *Caragana korshinskii*, a perennial native shrub species dominant in the region, were planted in the experimental plots. One-year-old seedlings were selected to improve establishment consistency among experimental plots under arid field conditions and to reduce variability associated with seed germination and very early establishment. After planting, seven precipitation treatments were established: −50%, −30%, −10%, control, +10%, +30%, and +50%, with three replicate plots per treatment and 21 plots in total. The planted shrubs were maintained in situ throughout the experimental period and were allowed to grow under the imposed precipitation treatments. No annual harvesting, clipping, or intentional removal of the shrubs was conducted during the experiment. Each plot measured 3 m × 3 m. For the precipitation-reduction treatments, a 3 m × 3 m steel frame was installed above the plot (Figure 8D). The frame was 2.0 m high on one side and 1.8 m high on the opposite side, forming a slight slope for rainwater collection. Transparent acrylic interception panels, each 10 cm wide, were mounted on the upper frame. Three, nine, and fifteen panels were used for the −10%, −30%, and −50% precipitation treatments, respectively, corresponding to the intended interception of 10%, 30%, and 50% of natural precipitation. Intercepted rainwater was collected and directed into a cylindrical barrel (50 cm in diameter and 50 cm in height) positioned beside the frame on a 1-m-high rectangular steel support. The collected water was conveyed through PVC pipes to the corresponding precipitation-addition plots. Each addition plot was equipped with a 3 m × 3 m water-distribution system consisting of 15 parallel perforated PVC pipes laid on the soil surface at approximately 20 cm intervals. Immediately after each natural precipitation event, collected rainwater equivalent to 10%, 30%, or 50% of the event precipitation was redistributed to the +10%, +30%, or +50% treatment plots, respectively. Control plots received ambient precipitation without interception or supplemental water. The experiment was initiated in 2011 and maintained through the 2025 growing season, representing 15 consecutive growing seasons of precipitation manipulation. Details of the precipitation treatments are summarized in Table 2.

### 4.3. Sample Collection and Analysis

#### 4.3.1. Plant Measurements

In August 2025, plant growth traits were measured for one *Caragana korshinskii* shrub in each experimental plot. Plant height was measured vertically from the soil surface to the highest point of the canopy. Two perpendicular crown diameters, representing the maximum crown width and its perpendicular width, were recorded for each shrub. Mean crown diameter was calculated as the average of the two measurements. The projected crown area (*A*) was estimated by approximating the shrub canopy as an ellipse:

*A* = π (*L*/2) (*W*/2)

where *L* and *W* represent the two perpendicular crown diameters. Estimated canopy cover of *C. korshinskii* was calculated as the projected crown area divided by the plot area (9 m^2^) and expressed as a percentage.

#### 4.3.2. Soil Sampling and Analyses

For the analyses reported in the present study, soil sampling was conducted in early September 2025, after 15 consecutive growing seasons of precipitation manipulation. In each plot, five soil cores were collected from the 0–20 cm soil layer using a systematic five-point sampling scheme, with one core collected from the center and four from the interior corners of the plot. The five soil cores from each plot were thoroughly homogenized to form one composite sample, resulting in a total of 21 composite soil samples. The samples were placed in sealed polyethylene bags and transported immediately to the laboratory. Each composite sample was divided into three portions. One portion was air-dried at room temperature for physicochemical and SOC-fraction analyses. A second portion was passed through a 2-mm sieve to remove visible roots, stones, and other coarse materials, homogenized, and stored at 4 °C for microbial biomass carbon, microbial biomass nitrogen, and extracellular enzyme activity analyses. The remaining portion was freeze-dried and stored at −80 °C until microbial community analysis.

Soil moisture content was determined gravimetrically by drying fresh soil samples at 105 °C for 12 h. Total soil organic carbon was determined using the potassium dichromate oxidation method with external heating [61]. SOC fractions were separated using a wet-sieving method. Briefly, 20 g of air-dried soil that had been previously passed through a 2-mm sieve was placed into a centrifuge tube, and 100 mL of 0.5% sodium hexametaphosphate solution was added to disperse the particles. The mixture was horizontally shaken at 180 rpm for 18 h to fully disperse soil aggregates [62]. The dispersed soil suspension was then passed through a 53-μm sieve and gently rinsed with deionized water. The material retained on the sieve (>53 μm) was collected as the POC fraction, whereas the fraction passing through the sieve (<53 μm) was considered the MAOC fraction [18]. Both fractions were dried at 60 °C to constant weight, weighed, and ground into fine powder. Organic carbon concentrations in the two fractions were determined using the potassium dichromate oxidation method with external heating, and the carbon contents of the >53 μm and <53 μm fractions were calculated as POC and MAOC, respectively.

#### 4.3.3. Microbial Biomass Carbon and Nitrogen

Microbial biomass carbon (MBC) and microbial biomass nitrogen (MBN) were quantified using the chloroform fumigation–extraction method [63,64]. Briefly, 20 g of fresh soil was divided into fumigated and non-fumigated subsamples. The fumigated subsamples were exposed to ethanol-free chloroform to lyse microbial cells, after which both fumigated and non-fumigated subsamples were extracted with 0.5 M K_2_SO_4_ solution. Extracted organic carbon was measured using a total organic carbon analyzer, and extracted nitrogen was determined via the Kjeldahl digestion method. MBC and MBN were calculated from the differences in extractable carbon and nitrogen between fumigated and non-fumigated samples using the extraction efficiency coefficient (*E* = 0.45):

MBC = (*C*_f_ − *C*_nf_)/*E*

MBN = (*N*_f_ − *N*_nf_)/*E*

where *C*_f_ and *C*_nf_ are the extractable carbon concentrations in the fumigated and non-fumigated samples, respectively, and *N*_f_ and *N*_nf_ are the corresponding extractable nitrogen concentrations.

#### 4.3.4. Soil Enzyme Activity

Soil extracellular enzyme activities were determined using fluorometric assays based on established methods [65]. β-Glucosidase (BG), *N*-acetyl-β-D-glucosaminidase (NAG), and phosphatase activities were measured as indicators of microbial C-, N-, and P-acquisition processes, respectively. Fluorescence signals were corrected for blank fluorescence and sample-specific fluorescence quenching, and enzyme activities were calculated using standard calibration curves and expressed as nmol g^−1^ h^−1^.

#### 4.3.5. Microbial Community Analysis

Soil microbial community composition was characterized by means of high-throughput sequencing of bacterial 16S rRNA genes and fungal internal transcribed spacer (ITS) regions. Total genomic DNA was extracted from 0.5 g of freeze-dried soil using the FastPure Soil DNA Isolation Kit (Magnetic Bead) (Shanghai Meiji Yuhua Biomedical Technology Co., Ltd., Shanghai, China) according to the manufacturer’s instructions. The integrity of the extracted DNA was assessed by agarose gel electrophoresis, and DNA concentration and purity were determined using a NanoDrop 2000 spectrophotometer (Thermo Fisher Scientific, Wilmington, DE, USA).

The V3–V4 region of the bacterial 16S rRNA gene was amplified using the primer pair 338F (5′-ACTCCTACGGGAGGCAGCAG-3′) and 806R (5′-GGACTACHVGGGTWTCTAAT-3′). The fungal ITS region was amplified using the primer pair ITS1F (5′-CTTGGTCATTTAGAGGAAGTAA-3′) and ITS2R (5′-GCTGCGTTCTTCATCGATGC-3′). PCR amplification was performed in a 20-μL reaction mixture containing 10 μL of 2× Pro Taq PCR Master Mix (Accurate Biology (Hunan) Co., Ltd., Changsha, Hunan, China), 0.8 μL of each primer, and approximately 10 ng of template DNA. The PCR program consisted of an initial denaturation at 95 °C for 3 min, followed by 27 cycles of denaturation at 95 °C for 30 s, annealing at 55 °C for 30 s, and extension at 72 °C for 45 s, with a final extension at 72 °C for 10 min.

PCR products were purified and quantified and subsequently subjected to paired-end sequencing on an Illumina NextSeq 2000 platform (Illumina, San Diego, CA, USA). Raw paired-end reads were quality-filtered, merged, and screened to remove chimeric sequences. The remaining high-quality sequences were clustered into operational taxonomic units (OTUs) at 97% sequence similarity. Taxonomic assignment was performed using appropriate reference databases, with the SILVA database (v138.2; https://www.arb-silva.de/ (accessed on 4 May 2026)) used for bacterial 16S rRNA sequences and the UNITE database (v10.0; https://unite.ut.ee/ (accessed on 4 May 2026)) used for fungal ITS sequences. Alpha-diversity indices, including Observed richness, Chao1 richness, Shannon diversity, Simpson index, and Pielou evenness, were calculated to evaluate microbial diversity across precipitation treatments.

For visualization of the phylogenetic relationships among dominant taxa, representative sequences of dominant OTUs were used to construct maximum-likelihood phylogenetic trees using FastTree (v2.2.0; https://morgannprice.github.io/fasttree/ (accessed on 4 May 2026)). The resulting phylogenetic trees were visualized using the Interactive Tree of Life (iTOL) platform (v7; https://itol.embl.de/ (accessed on 4 May 2026)), together with treatment-level relative abundance information.

### 4.4. Data Processing and Statistical Analyses

Daily precipitation data for 2011–2025 were obtained from the Gansu Linze National Field Scientific Observation and Research Station of Farmland Ecosystem, Chinese National Ecosystem Research Network (CNERN), using the dataset “Daily Values of Precipitation, Evaporation, and Visibility from Manual Atmospheric Observations.” Annual precipitation was calculated as the sum of daily precipitation within each calendar year. A precipitation day was defined as a day with precipitation > 0 mm, whereas a strong precipitation day was defined as a day with precipitation ≥ 5 mm. Maximum daily precipitation was defined as the largest daily precipitation amount recorded within each year. Annual precipitation anomalies were calculated relative to the mean annual precipitation during 2011–2025. Monthly precipitation totals were calculated from the daily records, and seasonal precipitation patterns were visualized using generalized additive models (GAMs). All analyses were performed in R version 4.5.1. Data processing and visualization used the *dplyr*, *tidyr*, *purrr*, *stringr*, *tibble*, *readxl*, *readr*, *ggplot2*, *scales*, *grid*, *agricolae* and *patchwork* packages. Normality and homogeneity of variance were assessed using the Shapiro–Wilk test and Levene’s test in the *car* package, respectively. Treatment effects on plant growth traits, SMC, SOC fractions, microbial biomass, enzyme activities, and microbial α-diversity were evaluated using one-way ANOVA followed by Tukey’s HSD test. Pearson correlations were used to assess relationships among variables. PCoAs based on Bray–Curtis dissimilarities and envfit analysis with 999 permutations were conducted using the *vegan* package. PLS-SEM was performed using *plspm* to quantify direct, indirect, and total effects linking precipitation, water availability, microbial attributes, enzyme activity, and carbon stability. Carbon stability was represented by SOC, POC, and the MAOC/POC ratio. Unless otherwise noted, statistical significance was defined at *p* < 0.05, and data are presented as means ± SE.

## 5. Conclusions

Based on a precipitation-manipulation experiment established in 2011 and comprehensively evaluated in 2025, this study shows that sustained changes in precipitation are associated with nonlinear responses in plant growth, microbial functioning, and SOC stabilization in an arid shrub ecosystem of China. Precipitation-induced variation in soil water availability was closely linked to microbial biomass and extracellular enzyme activity, which were, in turn, strongly associated with changes in SOC fractions. Bacterial communities were more sensitive to altered precipitation than fungal communities, whereas microbial α-diversity showed little direct association with carbon stability. These findings indicate that precipitation-driven SOC stabilization is more closely associated with microbial functional processes than with microbial taxonomic diversity. The weakening of plant growth, microbial functioning, and SOC accumulation under the highest precipitation addition suggests an apparent precipitation threshold beyond which the beneficial effects of additional water may diminish or reverse. Overall, microbial biomass accumulation and enzyme-mediated carbon transformation appear to represent important pathways linking precipitation variation to SOC stabilization in this dryland shrub ecosystem. Future research should integrate above- and belowground productivity, root-derived carbon inputs, root turnover, soil respiration, and long-term process measurements to clarify the interactions between plant carbon inputs and microbial regulation of SOC stability and to evaluate the persistence and broader applicability of this apparent precipitation threshold.

## Figures and Tables

**Table 1:** Summary of principal-coordinates analysis (PCoA) and envfit analyses for (a) bacterial and (b) fungal communities under altered precipitation.

**(a) Bacteria**				
**Parameter**	**PCoA1**	**PCoA2**	**R^2^**	***p* Value**
Eigenvalue	0.34	0.20	-	-
Cumulative percentage of variance (%)	25.25	39.67	-	-
BG	0.41	−0.43	0.36	0.02 *
AP	0.56	−0.09	0.32	0.03 *
NAG	0.37	−0.41	0.30	0.04 *
SOC	0.35	−0.39	0.28	0.04 *
POC	0.35	−0.39	0.27	0.04 *
MBN	0.47	−0.20	0.26	0.07
SMC	0.34	−0.36	0.25	0.08
MAOC/POC	0.47	−0.13	0.24	0.08
MBC	0.30	−0.26	0.16	0.21
**(b) Fungi**				
**Parameter**	**PCoA1**	**PCoA2**	**R^2^**	***p* Value**
Eigenvalue	1.02	0.50	-	-
Cumulative percentage of variance (%)	20.21	30.17	-	-
MBN	0.28	0.16	0.10	0.36
MAOC/POC	0.22	−0.20	0.09	0.41
BG	0.18	0.14	0.05	0.61
NAG	0.21	0.08	0.05	0.63
AP	0.16	0.03	0.03	0.79
SMC	−0.01	0.16	0.03	0.81
MBC	0.09	−0.03	0.01	0.92
SOC	0.08	0.04	0.01	0.93
POC	0.07	0.05	0.01	0.93

PCoA was conducted using Bray–Curtis dissimilarities calculated from microbial relative-abundance data. Environmental variables were fitted to the first two PCoA axes using the envfit function with 999 permutations. PCoA1 and PCoA2 values represent vector coordinates in the ordination space. Abbreviations: AP, phosphatase activity; BG, β-glucosidase activity; MAOC, mineral-associated organic carbon; MBC, microbial biomass carbon; MBN, microbial biomass nitrogen; NAG, N-acetyl-β-D-glucosaminidase activity; POC, particulate organic carbon; SMC, soil moisture content; SOC, soil organic carbon. R^2^ represents the squared correlation between each environmental variable and the ordination configuration, and *p* values were obtained from permutation tests. Significance level is *, *p* < 0.05.

**Table 2:** Precipitation treatments and details of the precipitation-manipulation experiment.

Treatment	Target Precipitation Relative to Ambient	Manipulation	Acrylic Panels	Replicates
−50%	50%	50% precipitation intercepted	15	3
−30%	70%	30% precipitation intercepted	9	3
−10%	90%	10% precipitation intercepted	3	3
Control	100%	Ambient precipitation	0	3
+10%	110%	+10% collected rainwater	—	3
+30%	130%	+30% collected rainwater	—	3
+50%	150%	+50% collected rainwater	—	3

Note: — indicates not applicable.

## Data Availability

The data supporting the findings of this study are available from the corresponding author upon reasonable request.

## Supplementary Materials

The following supporting information can be downloaded at https://www.mdpi.com/article/10.3390/plants15203090/s1. Figure S1: Phylogenetic distribution of dominant bacterial OTUs under altered precipitation; Figure S2: Phylogenetic distribution of dominant fungal OTUs under altered precipitation.
