# Supplementary material for: Precipitation Gradients Regulate Soil Carbon Stabilization Mediated by Microbes in an Arid Region of China
Source: Plants (Basel). 2026 Oct 9;15(20):3090. doi: 10.3390/plants15203090 (PMC13709077; doi:10.3390/plants15203090)
Supplement: Supplementary file 1 [file plants-15-03090-s001.zip › plants-4502119-supplementary.pdf]

(S1)

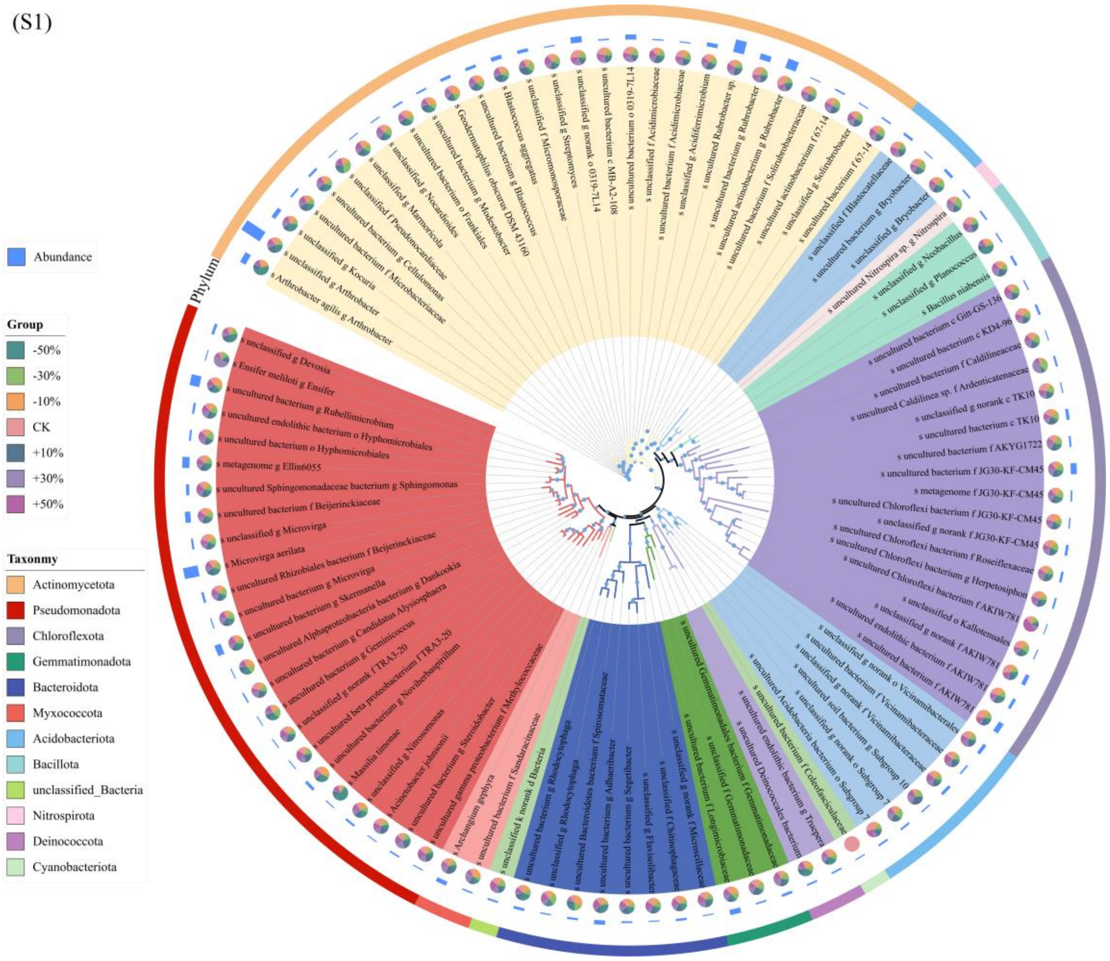

Figure S1. Phylogenetic distribution of dominant bacterial OTUs under altered precipitation.

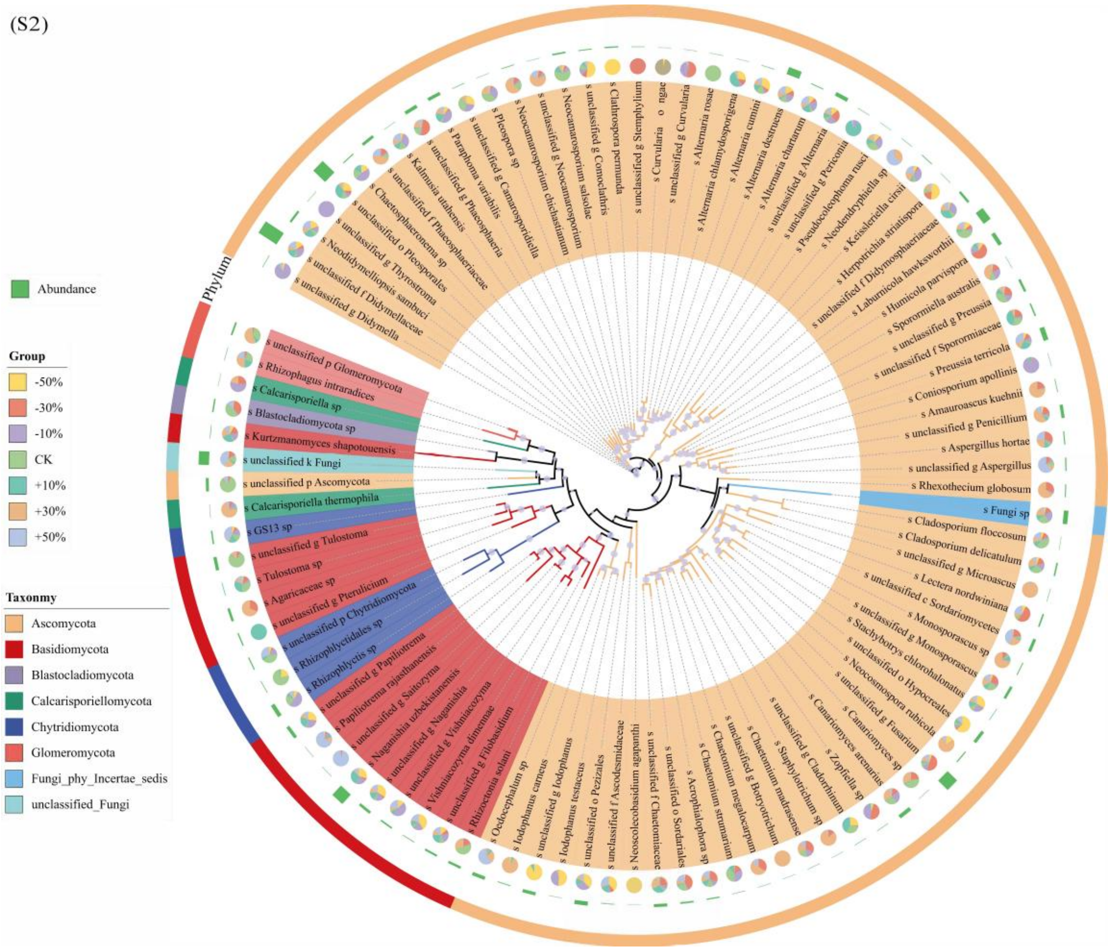

Figure S2. Phylogenetic distribution of dominant fungal OTUs under altered precipitation.
